# Clinicopathological and prognostic significance of PD-L1 and TIM-3 expression in medullary thyroid carcinoma: a retrospective immunohistochemistry study

**DOI:** 10.1007/s40618-023-02126-z

**Published:** 2023-07-18

**Authors:** D. Wusiman, L. Guo, L. Li, X. Zhang, X. Zhao, Z. An, Z. Huang, Y. Zhang, Z. Li, J. Ying, M. Wei, W. Li, C. An

**Affiliations:** 1 Department of Head and Neck Surgery, National Cancer Center/National Clinical Research Center for Cancer/Cancer Hospital, Chinese Academy of Medical Sciences and Peking Union Medical College, 100021 Beijing, China; 2 Department of Pathology, National Cancer Center/National Clinical Research Center for Cancer/Cancer Hospital, Chinese Academy of Medical Sciences and Peking Union Medical College, 100021 Beijing, China; 3 Department of Head and Neck Surgical Oncology, National Cancer Center/National Clinical Research Center for Cancer/Cancer Hospital, Shenzhen Hospital, Chinese Academy of Medical Sciences and Peking Union Medical College, 518116 Shenzhen, China

**Keywords:** Medullary thyroid carcinoma, Programmed death‐ligand 1 (PD-L1), T-cell immunoglobulin and mucin-domain containing-3 (TIM-3), Clinicopathologic factor, Prognosis

## Abstract

**Purpose:**

Expression of the programmed death-ligand 1 (PD-L1) and T-cell immunoglobulin and mucin-domain containing-3 (TIM-3) in medullary thyroid carcinoma (MTC) has been controversial and rarely reported.

**Methods:**

Surgical specimens of 190 MTC patients who had initial curative-intent surgery were collected. Immunohistochemistry of PD-L1 and TIM-3 was performed using 22C3 pharmDx (Dako, Carpinteria, CA) and anti-TIM-3 (1:500, ab241332, Abcam). Stained slides were scored using a combined positive score (CPS) with a cutoff of ≥ 1. We established correlations between PD-L1 expression, TIM-3 expression, clinicopathological, and survival data.

**Results:**

13 cases (13/190, 6.84%) were positive for PD-L1 expression, and 42 cases (42/154, 27.27%) for TIM-3 expression. PD-L1 expression was correlated to TIM-3 expression (*P* = 0.002), but was not related to overall survival (OS) or progression-free survival (PFS). TIM-3 expression was correlated to perineural invasion (*P* = 0.040). Multivariate Cox analysis showed that lymphovascular invasion (LVI) was independently associated with OS. And tumor size, LVI, and lymph node metastases were significantly associated with PFS. Furthermore, the multivariate logistic analysis showed multifocal status, LVI, pathological T stage and lymph node metastasis were independent risk factors for biochemical recurrence/persistent disease.

**Conclusions:**

We demonstrated that PD-L1 and TIM-3 expression were not frequent in MTC and were not associated with survival prognosis. Our results should be considered when clinical trials of PD-L1 or TIM-3 blockades are implemented.

**Supplementary Information:**

The online version contains supplementary material available at 10.1007/s40618-023-02126-z.

## Introduction

Medullary thyroid carcinoma is a rare malignant tumor deriving from parafollicular cells. Although MTC accounts for only 3–5% of all thyroids, it causes up to 13% of all thyroid cancer-related mortality [1, 2]. As the main treatment for localized MTC, surgical resection is often less effective in patients with metastasis or unresectable recurrence [3]. At present, two types of tyrosine kinase inhibitors (TKIs), vandetanib and cabozantinib, have significantly improved the progression-free survival (PFS) of patients, but failed to increase the overall survival (OS) rate [4, 5]. The application of recombinant yeast-CEA vaccines designed to stimulate an immune response against CEA in MTC has shown relatively low toxicity compared to TKIs for some patients with metastatic or recurrent MTC [6]. It indicated that vaccines did increase immune cells in the TME. However, the efficacy of the immune checkpoint blocker pembrolizumab (anti-PD-1) and other ICIs (immune checkpoint inhibitors) have not been investigated in patients with progressive MTC. The effect of immunotherapy targeted at immune checkpoints in treating MTC is still unclear, which urges us to explore the immune landscapes of MTC further.

PD-L1 is a ligand of programmed cell death 1 (PD-L1). As a member of inhibitory costimulatory signal molecules, PD-L1 plays a significant role in the immune evasion of tumor cells and the exhaustion of effector T cells [7]. Currently, the utilization of anti-PD-1 therapy to treat MTC is still a blank field. The expression level of PD-L1 in tumor cells and tumor-induced immune cells is still unclear, which limits the application of anti-PD-1 therapy to treat MTC. TIM-3 is one of the prevailing immune checkpoints after PD-1 and CTLA-4, which has four types of ligands, so there are many mechanisms involved in promoting immune evasion [8]. Immunotherapy for TIM-3 is still in clinical trials (NTC02608268, NTC02817633). As far as we know, only one study focused on the expression level of TIM-3 in MTC [9], and more studies are needed to confirm whether TIM-3 is suitable for the future treatment of MTC.

Our study performed immunohistochemistry in 190 MTC tissues to evaluate PD-L1 and TIM-3 expression levels in MTC and establish the correlation between their expression and MTC clinicopathological and survival factors. This study will provide valuable data and perspectives for immune checkpoint landscapes of MTC.

## Materials and methods

### Patients and samples

One hundred ninety cases receiving a histological diagnosis of MTC between August 2010 and December 2019 at Cancer Hospital, Chinese Academy of Medical Science, were retrospectively reviewed. The inclusion criteria included: (1) histologically confirmed MTC; (2) MTC without distant metastasis; (3) underwent initial surgery; (4) no previous treatment. The exclusion criteria were as follows: (1) a history of other tumors; (2) distant metastasis at diagnosis; (3) samples without available tissue for pathologic evaluation; (4) samples without sufficient clinical information. Clinical data collected included demographics, tumor characteristics, pathologic findings, prognosis, and survival time. This study was approved by our institutional review board of Cancer Hospital, Chinese Academy of Medical Sciences. Informed consent was waived for this retrospective study.

### Tissue microarray (TMA) construction

**Fig. 1:**
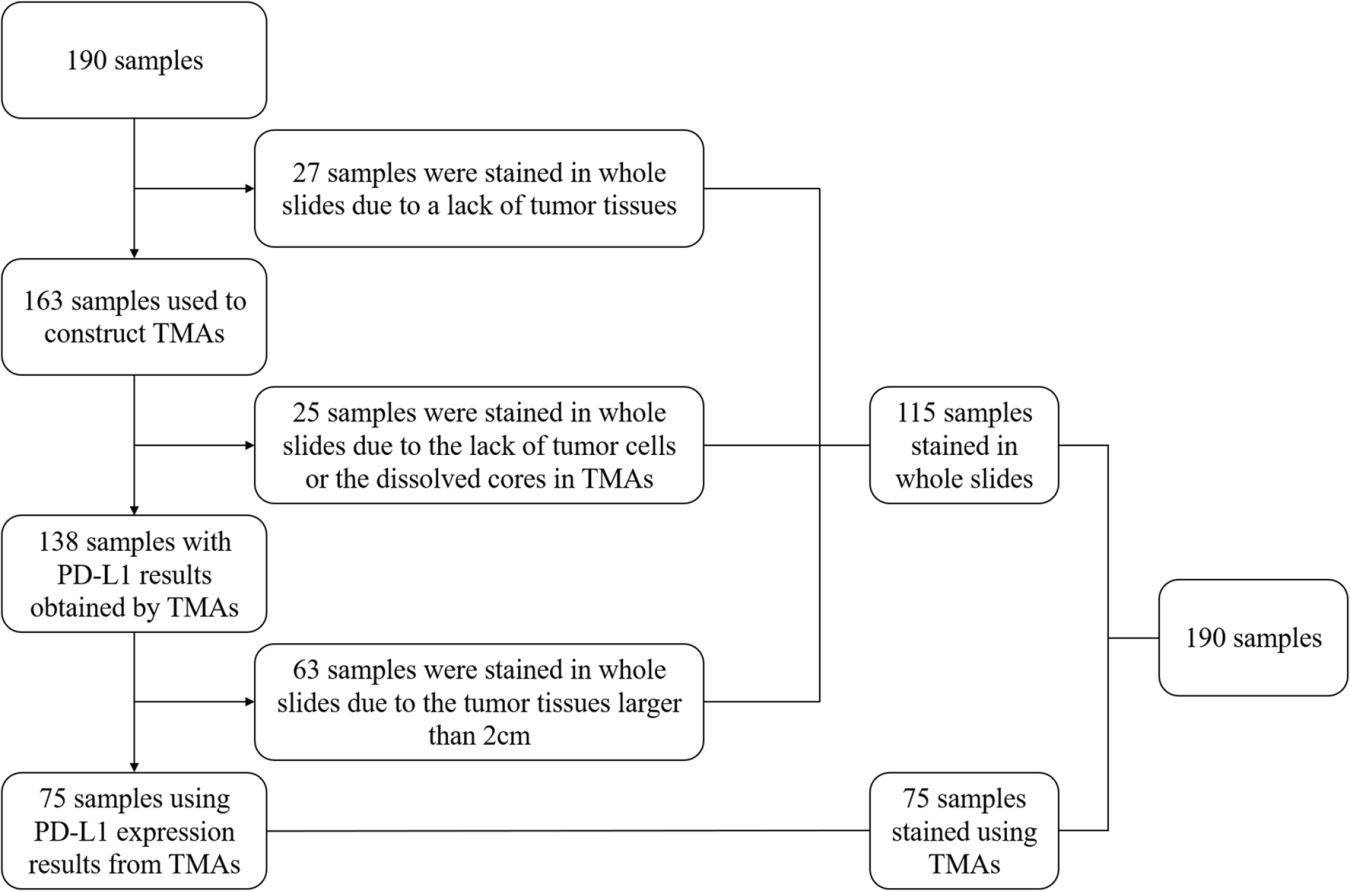
Workflow diagram of patient selection in tissue microarray construction to evaluate PD-L1 expression

We collected 190 formalin-fixed and paraffin-embedded (FFPE) tumor tissues. We used whole sections and TMAs of FFPE specimens in this study. Firstly, 27 tumor specimens were stained in whole slides due to a lack of tumor tissues. We constructed TMAs using the FFPE of the remaining 163 samples, and we stained 25 complete slides of surgical specimens because of the lack of tumor cells or the dissolved cores in TMAs. We have 138 cases with PD-L1 results obtained by TMAs. Considering that the TMAs results may not correctly respond to the IHC results of oversized tumor tissues, 63 samples larger than 2 cm were stained in whole slides. Finally, we used 75 cases where PD-L1 expression resulted from the TMAs. Among these 190 patients, 88 patients had both complete slides of surgical specimens results and TMAs results, and we used complete slides results for PD-L1 expression in these patients. We used the PD-L1 expression results of 115 whole slides and 75 core biopsies in TMAs. Among the TMAs with 163 samples, 154 cases were accessed using core biopsies of TMAs to analyze TIM-3 expression because of the lack of tumor cells in TMAs or the dissolved cores in nine patients. The flow chart for case screening was shown in Fig. 1. A dedicated head and neck pathologist marked representative tumor regions, and one 0.6 mm tissue core for each sample was obtained and arranged in the TMA using a manual tissue arrayer (Beecher Instruments Inc, Sun Prairie, WI, USA).

### Immunohistochemical staining and analysis

Immunostaining was performed with the following primary antibodies: 22C3 pharmDx (mouse monoclonal primary anti-PD-L1 antibody, prediluted, clone 22C3, Dako, Carpinteria, CA) and rabbit monoclonal [EPR22241] to TIM-3 (Abcam, ab241332, 1:200 dilutions). According to the manufacturer’s instructions, PD-L1 staining was used on the Autostainer Link 48 [10]. Pathological evaluation of PD-L1 and TIM-3 staining was according to earlier studies [9, 11]. It was assessed by combined positive score (CPS), defined as the number of positive tumor cells and immune cells divided by the total number of viable tumor cells multiplied by 100 [12], and CPS ≥ 1 was considered positive. Independent analysis of the IHC results was conducted by two pathologists blind to the patient information (L.L. and Y.Z.).

### Statistical analysis

For statistical analyses, R studio and R 4.2.0 were used. The two‐sided *P* value or Fisher’s exact tests was used to compare PD-L1 expression between different clinical factors as appropriate. Multivariate Cox proportional hazards regression models were established to evaluate the independent predictors of OS and PFS using the variables with significant influence in the univariate analysis. Multivariate logistic analyses were used to investigate the correlation between biochemical recurrence/persistent disease (BcR/BcPD) and clinicopathological factors. Kaplan–Meier survival curves and the Log-rank test were used to determine survival outcomes. Statistical significance was defined as a two‐sided *P* value ≤ 0.05.

## Results

### Clinicopathological characteristics of the patients

A total of 190 MTC were eligible for inclusion, including 93 males (48.9%) and 97 females (51.1%). The median age of the whole cohort at diagnosis was 48 years (range = 14–76 years). Most tumors were unifocal (76.8%), unilateral (80.5%), without lymphovascular invasion (LVI) (85.3%), and without perineural invasion (91.1%). The predominant cancer tumor stage was T1&T2 (67.9%), and most of them had lymph node metastasis (70.0%). The median follow-up time was 71.5 months (5–142 months). The characteristics of the patients are summarized in Table 1.

**Table 1:** Patient clinicopathologic characteristics during the study period (*N* = 190)

Characteristics	Overall cohort (*N* = 190)	PD-L1 negative (*N* = 177)	PD-L1 positive (*N* = 13)	*P*
TIM3 (%)				
Negative	112 (72.7)	111 (75.5)	1 (14.3)	**0.002**
Positive	42 (27.3)	36 (24.5)	6 (85.7)	
Age (%)				
< 60	148 (77.9)	139 (78.5)	9 (69.2)	0.489
≥ 60	42 (22.1)	38 (21.5)	4 (30.8)	
Sex (%)				
Female	97 (51.1)	93 (52.5)	4 (30.8)	0.157
Male	93 (48.9)	84 (47.5)	9 (69.2)	
Tumor size (%)				
≤ 20	127 (66.8)	118 (66.7)	9 (69.2)	1.000
> 20and ≤ 40	49 (25.8)	46 (26.0)	3 (23.1)	
> 40	14 (7.4)	13 (7.3)	1 (7.7)	
Multifocal (%)				
No	146 (76.8)	135 (76.3)	11 (84.6)	0.736
Yes	44 (23.2)	42 (23.7)	2 (15.4)	
Bilateral distribution (%)				
No	153 (80.5)	143 (80.8)	10 (76.9)	0.72
Yes	37 (19.5)	34 (19.2)	3 (23.1)	
Lymphovascular invasion (%)				
No	162 (85.3)	153 (86.4)	9 (69.2)	0.105
Yes	28 (14.7)	24 (13.6)	4 (30.8)	
Perineural invasion (%)				
No	173 (91.1)	160 (90.4)	13 (100.0)	0.611
Yes	17 (8.9)	17 (9.6)	0 (0.0)	
Pathological T stage (%)				
T1 and T2	129 (67.9)	119 (67.2)	10 (76.9)	0.555
T3 and T4	61 (32.1)	58 (32.8)	3 (23.1)	
Lymph node metastasis (%)				
No	57 (30.0)	54 (30.5)	3 (23.1)	0.758
Yes	133 (70.0)	123 (69.5)	10 (76.9)	
Pathologic TNM stage (%)				
I and II	62 (32.6)	59 (33.3)	3 (23.1)	0.552
III and IV	128 (67.4)	118 (66.7)	10 (76.9)	

Bold indicates *P* value less than 0.05

### The expression of PD-L1 and TIM-3 in MTC

**Fig. 2:**
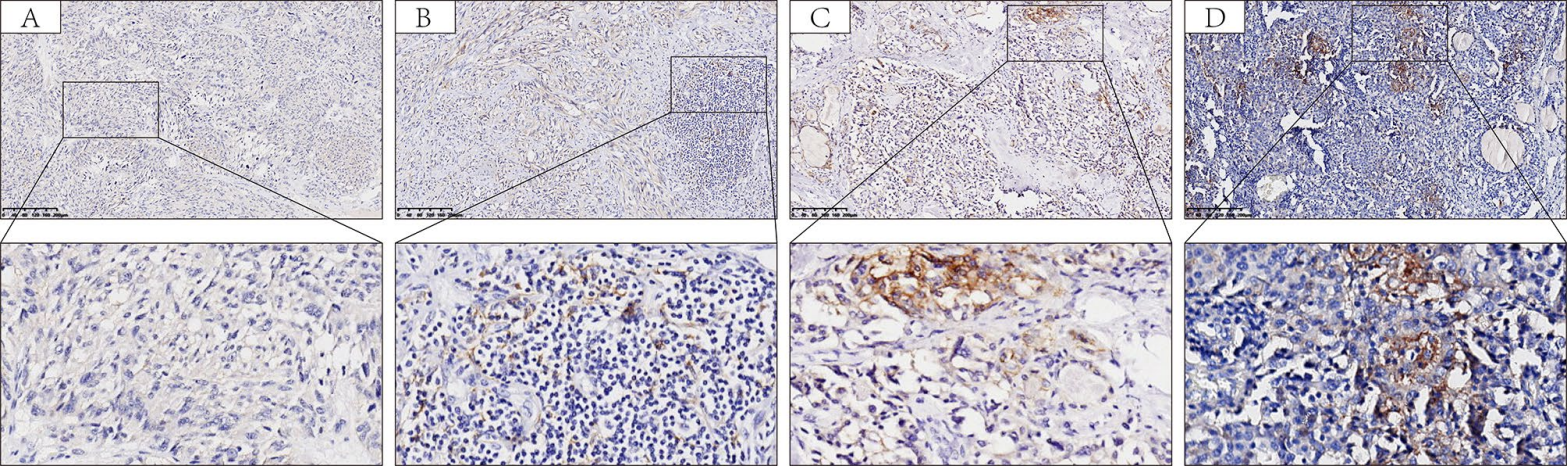
Representative low (100×) and high (400×) magnification immunohistochemistry images. **A** Negative PD‐L1 expression (CPS = 0) in the tumor tissue; **B** Low PD‐L1 expression (CPS = 1) in the tumor tissue; **C** Moderate PD-L1 expression (CPS = 5) in the tumor tissue; **D** High PD‐L1 expression (CPS = 10) in the tumor tissue

The PD-L1 positivity and TIM-3 positivity by CPS were observed in 13 (13/190, 6.8%) and 42 (42/154, 27.3%) patients, respectively (Fig. 2). Positive PD-L1 expression (CPS ≥ 1) was significantly correlated to positive TIM-3 expression (*P* = 0.002) using Fisher’s exact tests (Table 1). Perineural invasion (*P* = 0.040) was a significant factor associated with TIM-3 expression (Table S1). However, there was no correlation to other clinicopathological characteristics.

### Survival analysis of patients with MTC

In univariate analysis, PD-L1 positive (CPS ≥ 1) was not associated with OS (HR, 3.10; 95% CI, 0.68–14.20; *P* = 0.145) (Table S2) or PFS (HR, 1.20; 95% CI, 0.37–3.88; *P* = 0.765) compared with PD-L1 negative in the whole cohort (Table 2). And no prognostic impact of TIM-3 expression was noted, neither OS (HR, 1.46; 95% CI, 0.49–4.37; *P* = 0.502) (Table S2) nor PFS (HR, 1.48; 95% CI, 0.78–2.83; *P* = 0.232) (Table 2). We also analyzed the association between other clinical factors and survival. In univariate analysis, tumor size (*P* = 0.040) and LVI (*P* = 0.004) were significant factors associated with OS, and LVI status (*P* = 0.015) was confirmed as an independent prognostic parameter for OS in different groups using multivariate analyses (Table S2).

**Table 2:** Univariate and multivariate Cox analysis for progression-free survival in patients with medullary thyroid carcinoma (*N* = 186)

Variables	Progression-free survival			
	Univariate		Multivariate	
	HR (95%CI)	*P*	HR (95%CI)	*P*
PD-L1				
Negative	Reference			
Positive	1.20 (0.37–3.88)	0.765		
TIM3				
Negative	Reference			
Positive	1.48 (0.78–2.83)	0.232		
Age				
< 60	Reference			
≥ 60	1.01 (0.5–2.03)	0.987		
Sex				
Female	Reference			
Male	1.29 (0.71–2.32)	0.399		
Tumor size				
≤ 20	Reference		Reference	
> 20 and ≤ 40	2.21 (1.14–4.26)	**0.018**	1.53 (0.77–3.05)	0.226
> 40	5.68 (2.57–12.54)	** < 0.001**	3.73 (1.61–8.63)	**0.002**
Multifocal				
No	Reference		Reference	
Yes	2.00 (1.08–3.68)	**0.027**	1.68 (0.90–3.15)	0.106
Bilateral distribution				
No	Reference			
Yes	1.31 (0.65–2.65)	0.454		
Lymphovascular invasion				
No	Reference		Reference	
Yes	3.77 (2.02–7.05)	** < 0.001**	2.10 (1.07–4.11)	**0.031**
Perineural invasion				
No	Reference			
Yes	2.09 (0.97–4.5)	0.060		
Pathological T stage				
T1 and T2	Reference			
T3 and T4	3.93 (2.13–7.26)	** < 0.001**		
Lymph node metastasis				
No	Reference		Reference	
Yes	7.13 (2.21–23)	**0.001**	4.61 (1.39–15.29)	**0.013**
Pathologic TNM stage				
I and II	Reference			
III and IV	4.51 (1.78–11.43)	**0.002**		

Bold indicates *P* value less than 0.05

Whereas tumor size (*P* = 0.018), multifocal status (*P* = 0.027), LVI (*P* < 0.001), pathological T stage (*P* < 0.001), lymph node metastasis (*P* = 0.001), and pathologic TNM stage (*P* = 0.002) were significantly associated with PFS using univariate analysis (Table 2). We carefully incorporated parameters without repeated information so that no over-adjustment would occur. We excluded the TNM stage in multivariate analyses, considering it contains information on tumor size, pathological T stage, and lymph node metastasis status. Tumor size (*P* = 0.002), LVI status (*P* = 0.031), and lymph node metastasis (*P* = 0.013) were confirmed as independent prognostic parameters for PFS (Table 2).

**Fig. 3:**
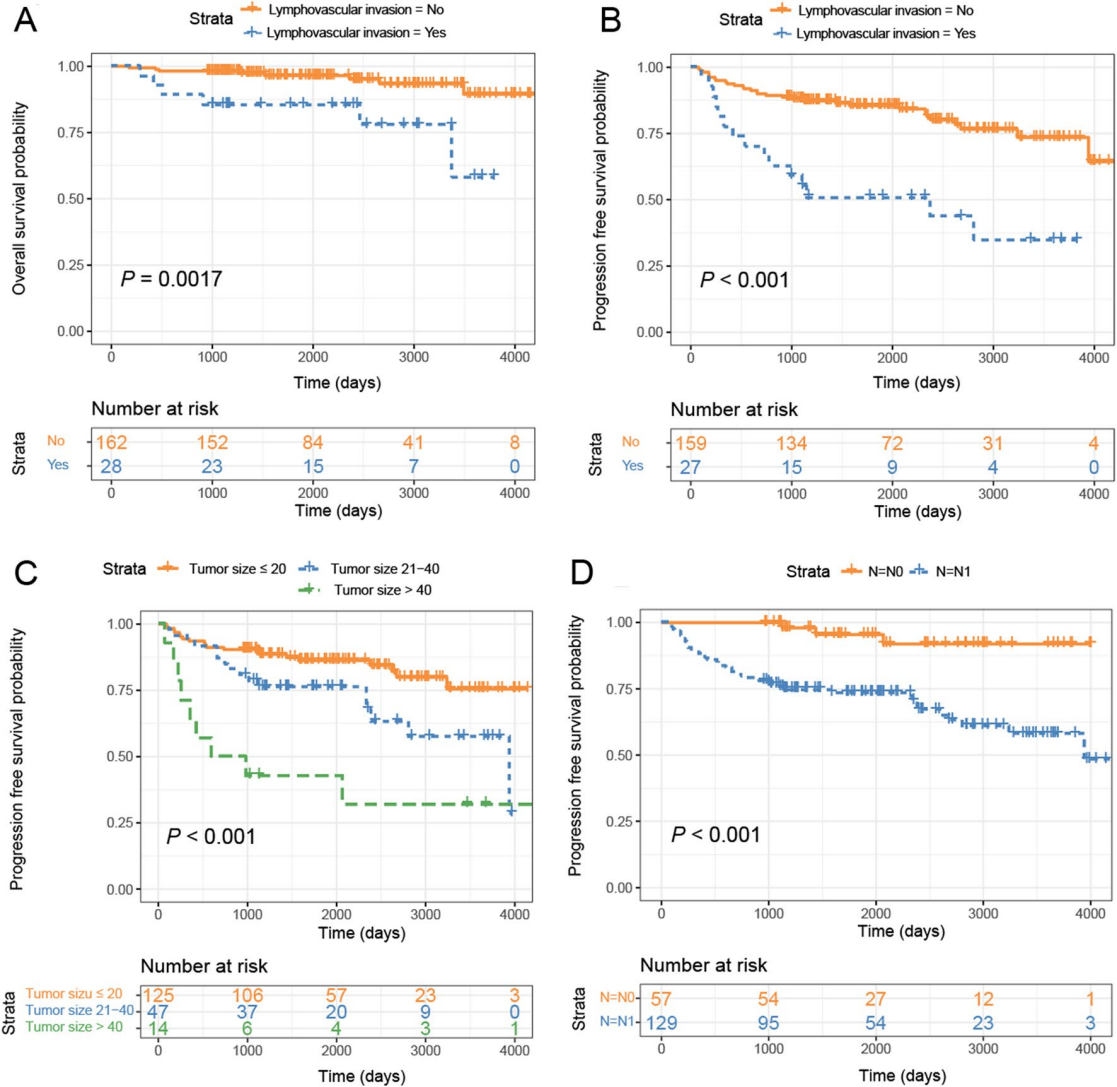
Kaplan–Meier analysis of the patients with medullary thyroid carcinoma. **A** Overall survival that stratified by lymphovascular invasion; **B** Progression-free survival that stratified by lymphovascular invasion; **C** Progression-free survival that stratified by tumor size; **D** Progression-free survival that stratified by N stage

Kaplan–Meier OS analysis revealed that MTC patients with LVI showed a shorter OS time than those without LVI (*P* = 0.0017) (Fig. 3A). A PFS advantage was observed for MTC patients without LVI, or tumor size ≤ 20, or without lymph node metastasis (Fig. 3B–D). Due to the lack of patients with positive PD-L1 expression, we only test TIM-3 in our study for the prognostic significance in patients with various clinicopathological factors.

### Correlation between clinicopathological factors and BcR/BcPD of MTC patients

Previous studies have found that the 10-year survival rate for biochemically cured MTC patients is 97.7% [13]. Hence, the association between BcR/BcPD and clinicopathological factors was analyzed by logistic regression analyses. We did not find the correlation between PD-L1 expression level and BcR/BcPD. We found some clinical characteristics that were significant risk factors of BcR/BcPD, including sex, tumor size, multifocal status, LVI, perineural invasion, pathologic T stage, lymph node metastasis, and pathologic TNM stage (*P* < 0.05) (Table 3). Multivariate logistic regression analyses indicated that patients with multifocal status, LVI, pathologic T stage, and lymph node metastasis are prone to suffer a high risk of BcR/BcPD (*P* < 0.05) (Table 3).

**Table 3:** Univariate and multivariate logistic analysis for biochemical recurrence/persistent disease in patients with medullary thyroid carcinoma (*N* = 173)

Variables	Biochemical recurrence/persistent disease			
	Univariate		Multivariate	
	OR (95%CI)	*P*	OR (95%CI)	*P*
PD-L1				
Negative	Reference			
Positive	0.92 (0.3–2.87)	0.891		
TIM3				
Negative	Reference			
Positive	1.4 (0.65–3.02)	0.390		
Age				
< 60	Reference			
≥ 60	1.69 (0.81–3.56)	0.165		
Sex				
Female	Reference		Reference	
Male	1.86 (1.02–3.41)	**0.043**	1.67 (0.74–3.84)	0.217
Tumor size				
≤ 20	Reference			
> 20and ≤ 40	3.09 (1.5–6.39)	**0.002**		
> 40	4.8 (1.23–18.68)	**0.024**		
Multifocal				
No	Reference		Reference	
Yes	3.86 (1.78–8.38)	**0.001**	3.65 (1.42–10.03)	**0.009**
Bilateral distribution				
No	Reference			
Yes	0.96 (0.45–2.02)	0.905		
Lymphovascular invasion				
No	Reference		Reference	
Yes	7.75 (2.54–23.64)	** < 0.001**	2.76 (0.86–10.82)	**0.109**
Perineural invasion				
No	Reference		Reference	
Yes	15.04 (1.91–118.46)	**0.010**	7.37 (1.19–144.38)	0.072
Pathological T stage				
T1 and T2	Reference		Reference	
T3 and T4	6.66 (3.16–14.02)	** < 0.001**	3.28 (1.40–8.08)	**0.008**
Lymph node metastasis				
No	Reference		Reference	
Yes	25.83 (8.7–76.63)	** < 0.001**	10.48 (3.61–38.39)	** < 0.001**
Pathologic TNM stage				
I and II	Reference			
III and IV	12.26 (5.29–28.39)	** < 0.001**		

Bold indicates *P* value less than 0.05

## Discussion

The application of ICIs has successfully improved the therapeutic effect of several malignant tumors and prolonged the prognosis of patients [14–16]. However, attempts in MTC are not sufficient. One of the reasons is that we have not yet explained the expression levels of MTC immune checkpoints and their correlation with clinicopathologic features and prognosis signatures clearly. Our study provided enough data to study the expression profiles of PD-L1 and TIM-3 in MTC.

In this study, the positive rate of PD-L1 expression in MTC was 6.84% (13 /190). MTC has a low positive rate of PD-L1 expression compared with other solid tumors, generally ranging from 12.2% to 32% (Table 4) [9, 17–22]. We observed heterogeneity among these studies mainly due to the different sample sizes. Only two articles from Bi Y and Shi X's groups had a study sample size of > 50 cases, and the PD-L1 expression ratios were 21.8% (19/87) and 14.4% (29/201), respectively. Shi’s study used clone 22C3, similar to ours, and Bongiovanni, Bi, and Kemal used clone SP263. Although some studies showed 22C3 PharmDx assay and SP263 assay could be used to identify PD-L1-positive cases and show high agreement [10, 23, 24], a study showed 22C3 assay and SP263 assay had significant discrepancies [25]. The study by Fonseca et al. used both 22C3 and SP142 antibodies and found PD-L1 expression rates of 75% and 0. However, there are major problems with this experiment, as its sample size was only six cases [18]. And it is still unknown whether there will be heterogeneity in using different antibodies in the MTC.

**Table 4:** PD-L1 expression in medullary thyroid carcinoma using IHC

Study	Year	Country	Samples	Follow-up Range (months)	Antibody	Scoring system	Positivity rate	Survival Parameter	Clinical Parameter
Bongiovanni et al.	2017	Switzerland	16	NS	SP263	CPS > 1	3/16 (18.8%)	Negative finding	Negative finding
Bi et al.	2019	China	87	15.7–72.4 (mean = 37.7)	SP263	CPS > 1	19/87 (21.8%)	Negative findings with OS and PFS	Correlated with distant metastases at the surgery
Shi et al.	2019	China	201	2–153 (mean = 73)	22C3	CPS > 1	29/201 (14.4%)	Worse 5-year SRFS	Correlated with tumor size, LNM, TNM staging, SR and BcR/BcPD
Pozdeyev et al.	2020	USA	41	NS	28-8	H	13/41(32%)	NS	NS
Fonseca et al.	2021	Portugal	8	NS	22C3; SP142	CPS > 1; stained cells > 1%	6/8(75%); 0	NS	NS
Kemal et al.	2022	Turkey	41	15–127 (mean = 54)	SP263	TPS > 1%	5/41(12.2%)	Negative finding	Negative finding

*IHC* immunohistochemistry; *NS* not stated; *CPS* combined positive score; *TPS* tumor proportion score; *H H*-Score; *OS* overall survival; *SRFS* structural recurrence-free survival; *SR* structural recurrence; *BcR/BcPD* biochemical recurrence/persistent disease; *LNM* lymph node metastasis

Bongiovanni et al. and Kemal et al. found no evident correlation between PD-L1 expression and clinicopathological factors or survival. Their findings were consistent with ours. Bi et al. showed PD-L1 correlated with distant metastases at surgery, but other studies excluded patients with distant metastasis at diagnosis, so we cannot verify this. But there is evidence that PD-L1 is highly expressed in MTC patients with incurable recurrence (8 /20, 40%) in Shi’s study [22]. We further analyzed the correlation between other clinicopathological factors and survival. OS was related to tumor size and LVI. And tumor size, LVI status, and lymph node metastasis were independent prognostic parameters of PFS, consistent with tumor biological behavior.

Besides, we explored the clinicopathological factors correlated with BcR/BcPD. And multifocal status, LVI, pathologic T stage, and lymph node metastasis appeared to be the most potent factors indicating BcR/BcPD, whether in univariate or multivariate logistic regression analyses. Our result corresponded with the survival and long-term biochemical cure study in MTC in Denmark, which suggested that the absence of regional lymph node metastases is the only independent prognostic factor for long-term biochemical cure [26]. It’s a pity that we failed to find the correlation between PD-L1 expression level and BcR/BcPD, and it might be attributed to the low positive rate of PD-L1. In the study of Shi et al., PD‐L1 positivity was independently associated with BcR/BcPD (*P* = 0.025), which might represent more clinical value [22].

Shi et al. reported that PD-L1 was coexpressed in 44.4% (12 /27) of PD-1 positive tumors [9], which means that PD-L1 is strongly related to the expression of PD-1. MTC patients with PD-L1 and PD-1 coexpression are likely to become potential targets for anti-PD-1 therapy. Whether PD-1 /PD-L1 coexpression predicts a more severe prognosis is still unknown [9, 22], so the influence of anti-PD-1 treatment in MTC prognosis needs to be verified by more clinical trials. In addition to PD-1, TIM-3 has been a new direction of immunotherapy in many clinical trials [27, 28]. TIM-3 is expressed in various T cells and myeloid cells [29]. Many studies have shown that the high expression of TIM-3 is related to the poor prognosis of patients [30–32]. Separated from previous studies, we found that perineural invasion is an essential factor associated with TIM-3 expression. Perineural invasion is the channel of intracranial extension of head and neck tumors. It indicates that perineural invasion reduces the survival rate, increases the local recurrence rate, and shortens the recurrence time [33]. In addition, we found the positive expression of PD-L1 is significantly related to the positive expression of TIM-3. And alternative up-regulation of TIM-3 will occur in lung adenocarcinoma patients treated with anti-PD-1 therapy [34]. Although PD-1/PD-L1 ICIs have improved the prognosis of patients with some solid tumor types, follow-up treatment for patients with primary and acquired resistance has always been troubling. To address this problem, researchers are exploring combinations of ICIs to expand the beneficiary population, including regimens with new immune checkpoints (e.g., TIM-3) [35]. Therefore, analyzing the expression of TIM-3 in MTC helps us to explore the possibility of implementing combinations of ICIs (e.g., PD-L1 and TIM-3). In our study, the positive rate of TIM-3 expression in MTC was 27.3% (42/154). The efficacy of TIM-3 blocking in the treatment of MTC requires further investigation.

In addition to our research, other immune checkpoint molecules’ expression in MTC was rarely studied. In the study of Shi et al., the positive rates and clinical significance of CTLA-4, LAG-3, and TIGIT in MTC were also reported [9]. The positive rate of CTLA-4 increased with the progress of the TNM stage and was associated with poor structural recurrence-free survival (SRFS). In contrast, the positive rates of LAG-3 and TIGIT were lower than CTLA-4 and had no clear correlation with the clinical characteristics of patients.

Our study has several limitations. Firstly, patients received surgery rather than immunotherapy. And this study is not a clinical validation study, so we could not analyze the predictive value of PD-L1 expression on immunotherapy assessment. Secondly, almost forty percent of the samples used TMA instead of whole tissue slides, which could potentially And samples with tumor diameters greater than 2 cm were stained for complete slides to prevent deviant CPS scores due to tumor heterogeneity.

## Conclusions

In conclusion, based on the current MTC research results, we have a preliminary understanding of the expression of PD-L1 and TIM-3 in MTC. Although no prognostic significance of PD-L1 or TIM-3 expression was observed in MTC patients, we found some clinicopathological characteristics related to OS, PFS, and BcR/BcPD in MTC patients.

### Supplementary Information

Below is the link to the electronic supplementary material.

Supplementary file1 (DOCX 27 KB)

## Data Availability

All data is available upon reasonable request to the corresponding author.
