# Supplementary material for: Clinicopathological and prognostic significance of PD-L1 and TIM-3 expression in medullary thyroid carcinoma: a retrospective immunohistochemistry study
Source: J Endocrinol Invest. 2023 Jul 18;47(1):91–100. doi: 10.1007/s40618-023-02126-z (PMC10776706; doi:10.1007/s40618-023-02126-z)
Supplement: Supplementary file 1 — Supplementary file1 (DOCX 27 KB) [file 40618_2023_2126_MOESM1_ESM.docx]

**Supplementary Materials**

| **Table S1** Patient clinicopathologic characteristics and events during the study period (N=154) | | | | | |
| --- | --- | --- | --- | --- | --- |
| Characteristics | | Overall Cohort (N=154) | TIM-3 negative (N=112) | TIM-3 positive (N=42) | *P* |
| **PD-L1 (%)** | Negative | 147 (95.5) | 111 (99.1) | 36 (85.7) | **0.002** |
|  | Positive | 7 (4.5) | 1 (0.9) | 6 (14.3) |  |
| **Age (%)** | <60 | 121 (78.6) | 91 (81.2) | 30 (71.4) | 0.193 |
|  | ≥60 | 33 (21.4) | 21 (18.8) | 12 (28.6) |  |
| **Sex (%)** | Female | 80 (51.9) | 57 (50.9) | 23 (54.8) | 0.719 |
|  | Male | 74 (48.1) | 55 (49.1) | 19 (45.2) |  |
| **Tumor size (%)** | ≤20 | 93 (60.4) | 68 (60.7) | 25 (59.5) | 0.889 |
|  | >20and≤40 | 47 (30.5) | 33 (29.5) | 14 (33.3) |  |
|  | >40 | 14 (9.1) | 11 (9.8) | 3 (7.1) |  |
| **Multifocal (%)** | No | 115 (74.7) | 81 (72.3) | 34 (81.0) | 0.306 |
|  | Yes | 39 (25.3) | 31 (27.7) | 8 (19.0) |  |
| **Bilateral distribution (%)** | No | 121 (78.6) | 89 (79.5) | 32 (76.2) | 0.664 |
|  | Yes | 33 (21.4) | 23 (20.5) | 10 (23.8) |  |
| **Lymphovascular invasion (%)** | No | 129 (83.8) | 95 (84.8) | 34 (81.0) | 0.625 |
|  | Yes | 25 (16.2) | 17 (15.2) | 8 (19.0) |  |
| **Perineural invasion (%)** | No | 138 (89.6) | 104 (92.9) | 34 (81.0) | **0.040** |
|  | Yes | 16 (10.4) | 8 (7.1) | 8 (19.0) |  |
| **Pathological T stage (%)** | T1&T2 | 98 (63.6) | 73 (65.2) | 25 (59.5) | 0.574 |
|  | T3&T4 | 56 (36.4) | 39 (34.8) | 17 (40.5) |  |
| **Lymph node metastasis (%)** | No | 41 (26.6) | 33 (29.5) | 8 (19.0) | 0.224 |
|  | Yes | 113 (73.4) | 79 (70.5) | 34 (81.0) |  |
| **Pathologic TNM stage (%)** | I&II | 43 (27.9) | 35 (31.2) | 8 (19.0) | 0.160 |
|  | III&IV | 111 (72.1) | 77 (68.8) | 34 (81.0) |  |

| **Table S2** Univariate Cox analysis for Overall survival in patients with medullary thyroid carcinoma (N=190) | | | | | | |
| --- | --- | --- | --- | --- | --- | --- |
| Variables | | Overall survival | | | | |
|  |  | Univariate | |  | Multivariate | |
|  |  | HR(95%CI) | *P* |  | HR(95%CI) | *P* |
| **PD-L1** | |  |  |  |  |  |
|  | Negative | reference |  |  |  |  |
|  | Positive | 3.10 (0.68 - 14.20) | 0.145 |  |  |  |
| **TIM3** | |  |  |  |  |  |
|  | Negative | reference |  |  |  |  |
|  | Positive | 1.46 (0.49 - 4.37) | 0.502 |  |  |  |
| **Age** |  |  |  |  |  |  |
|  | <60 | reference |  |  |  |  |
|  | ≥60 | 1.89 (0.63 - 5.64) | 0.256 |  |  |  |
| **Sex** |  |  |  |  |  |  |
|  | Female | reference |  |  |  |  |
|  | Male | 1.29 (0.45 - 3.73) | 0.637 |  |  |  |
| **Tumor size** | |  |  |  |  |  |
|  | ≤20 | reference |  |  | reference |  |
|  | >20and≤40 | **3.14 (1.05 - 9.34)** | **0.040** |  | 2.32 (0.75 - 7.23) | 0.145 |
|  | >40 | 1.30 (0.16 - 10.9) | 0.808 |  | 0.83 (0.10 - 7.26) | 0.869 |
| **Multifocal** | |  |  |  |  |  |
|  | No | reference |  |  |  |  |
|  | Yes | 2.55 (0.89 - 7.37) | 0.083 |  |  |  |
| **Bilateral distribution** | | |  |  |  |  |
|  | No | reference |  |  |  |  |
|  | Yes | 1.17 (0.33 - 4.19) | 0.811 |  |  |  |
| **Lymphovascular invasion** | | |  |  |  |  |
|  | No | reference |  |  | reference |  |
|  | Yes | **4.68 (1.62 - 13.50)** | **0.004** |  | **3.99 (1.31 - 12.14)** | **0.015** |
| **Perineural invasion** | |  |  |  |  |  |
|  | No | reference |  |  |  |  |
|  | Yes | 1.44 (0.32 - 6.46) | 0.634 |  |  |  |
| **T stage** | |  |  |  |  |  |
|  | T1&T2 | reference |  |  |  |  |
|  | T3&T4 | 1.65 (0.58 - 4.75) | 0.351 |  |  |  |
| **Lymph node metastasis** | | |  |  |  |  |
|  | No | reference |  |  |  |  |
|  | Yes | 5.29 (0.69 - 40.48) | 0.109 |  |  |  |
| **Pathologic TNM stage** | | |  |  |  |  |
|  | I&II | reference |  |  |  |  |
|  | III&IV | 5.87 (0.77 - 44.95) | 0.088 |  |  |  |
